# Supplementary material for: Predictors of treatment failure during the first year in newly diagnosed type 2 diabetes patients: a retrospective, observational study
Source: PeerJ. 2021 Mar 2;9:e11005. doi: 10.7717/peerj.11005 (PMC7934644; doi:10.7717/peerj.11005)
Supplement: Table S1 — If the value of VIF is less than 2, the multicollinearity problem is considered absent. [file peerj-09-11005-s002.docx]

| **Variables** | **All variables** | | **Primary model** | |
| --- | --- | --- | --- | --- |
|  | **Tolerance** | **VIF** | **Tolerance** | **VIF** |
| Age at onset | 0.707 | 1.414 | 0.737 | 1.357 |
| Gender | 0.734 | 1.362 | 0.777 | 1.287 |
| Level of education | 0.654 | 1.530 | 0.667 | 1.500 |
| Family history of DM | 0.913 | 1.095 |  |  |
| Current smoking | 0.803 | 1.246 | 0.810 | 1.235 |
| Physical activity | 0.904 | 1.106 | 0.913 | 1.095 |
| Medication adherence | 0.991 | 1.009 | 0.993 | 1.007 |
| Knowledge regarding GC | 0.657 | 1.523 |  |  |
| Willingness toward DSM | 0.949 | 1.054 |  |  |
| Perform SMBG | 0.781 | 1.280 | 0.774 | 1.292 |
| HbA1c at baseline | 0.905 | 1.105 | 0.931 | 1.074 |
| Mean BP | 0.958 | 1.044 | 0.964 | 1.037 |
| Total cholesterol | 0.653 | 1.532 |  |  |
| Triglycerides | 0.599 | 1.670 | 0.941 | 1.063 |
| HDL-C | 0.713 | 1.403 |  |  |
| Anti-diabetic Medication | 0.893 | 1.119 | 0.899 | 1.112 |
| Use of fibrates | 0.771 | 1.298 |  |  |
| Enrollment time | 0.597 | 1.675 | 0.760 | 1.316 |
